# Editorial: Women in developmental psychopathology and mental health

**DOI:** 10.3389/frcha.2025.1556534

**Published:** 2025-04-15

**Authors:** Isabelle V. Daignault, Eva Mohler

**Affiliations:** ^1^ École de Criminologie, Faculté des Arts et des Sciences, Université de Montréal, Montréal, Canada; ^2^ Clinic for Child and Adolescent Psychiatry, Psychosomatics and Psychotherapy, Saarland University Hospital, Homburg, Germany

**Keywords:** women, developmental psychopathology, trauma, violence, research

Editorial on the Research Topic Women in developmental psychopathology and mental health

As outlined by Frontiers in their invitation to this special issue collection, *Women in Developmental Psychopathology and Mental Health*, the United Nations Educational, Scientific and Cultural Organization (UNESCO) Institute for Statistics (June 2019) reported that women currently make up less than 30% of researchers worldwide. These numbers range from 18.2% to 48.2% in Central Asia and 32.7% in North America and Central Europe. This stark figure reflects the persistent gender gap in science, which is perpetuated by historical biases and enduring stereotypes. Even among researchers with similar careers and records, disparities persist (1), extending to gender imbalances in the world's most prestigious international research awards (2).

While social sciences (e.g., social work, education, speech-language therapy) have reached parity and, in some cases, become female-dominated in author-gender ratios, Holman et al. (3) observed that the gender gap remains striking in Science, Technology, Engineering, Mathematics, and Medicine (STEMM). They estimated that men are 1.7–2.1 times more likely than women to be invited to submit papers in these fields. These barriers create significant challenges for women pursuing scientific careers while also limiting the recognition of their invaluable contributions to research.

Recognizing and celebrating women's scientific achievements is crucial—not only for fostering recognition but also for inspiring future generations of scientists. As underscored by UNESCO, gender equality in science is a key driver of sustainable development. Breaking traditional barriers requires active efforts to promote gender parity, dismantle stereotypes, and ensure greater visibility and support for women in scientific fields.

In recent years, there has been growing acknowledgment of the pivotal role women researchers play in advancing the fields of developmental psychopathology and mental health. Considering that women represent the vast majority of caregivers for children's and elders' mental health (4), their contributions have been instrumental in shaping our understanding of trauma and the challenges faced by vulnerable populations. As argued by Judith Herman (5), whose work itself is an example of how women have shaped trauma studies, the recognition of trauma is deeply tied to feminist activism and to the contributions of female clinicians and researchers who worked with survivors of intimate violence.

This Research Topic is dedicated to showcasing insights from women researchers working at the intersection of trauma and developmental psychopathology, emphasizing research that addresses these challenges across diverse populations.

## The vision of this Research Topic

It was with this vision that Dr. Eva Möhler and I accepted the invitation to act as co-editors of this Research Topic. Our mission was to create a platform to promote and amplify the work of women scientists across developmental psychopathology and mental health. To ensure this focus, we invited submissions where the first or last author identifies as a woman, highlighting the diversity of research being conducted. Despite the broad scope of developmental psychopathology, a unifying theme emerged: trauma-related issues. Trauma remains a critical focus in the field, with profound implications for children, adolescents, caregivers, and mental health professionals alike. The contributions in this issue explore trauma-related challenges through multiple lenses, offering unique insights into the systemic, interpersonal, and psychological factors that shape mental health outcomes.

## Highlights of the collection

### Therapy non-completion for children with sexual behavior problems

The collection begins with a critical investigation into barriers to effective intervention for children with sexual behavior problems. The study sheds light on the challenges hindering therapy completion and highlights actionable pathways to improve outcomes for this underserved group within mental health systems. Notably, the findings emphasize the importance of placement stability, social support networks, and maternal support in facilitating therapy completion and enhancing the likelihood of successful intervention.

### Child maltreatment among autistic children in protection services

Another study explores the complexities of child maltreatment among autistic children involved in child protection services. Results revealed that autistic children appear at higher risk of child maltreatment and at higher risk of presenting co-occurring conditions compared to their non-autistic peers. By examining the specific forms and correlates of maltreatment, the research underscores the need for tailored interventions and robust support systems to address the unique challenges faced by this vulnerable population.

### Residential child care worker and perceptions of well-being and work-related stress

Brend et al. examine the well-being of residential childcare workers by exploring their sense of work-related pride and achievement. Given that caretakers' attitudes play a crucial role in the success of treatment in residential mental health facilities, this study addresses an important aspect of child welfare. It identifies a rarely studied protective mechanism for childcare workers, which relies on the reciprocity, trust, and confidence established in their relationships with children. Their findings are relevant not only to mental health professionals but also to child caretakers in daycare settings, emphasizing the importance of prevention in child mental health.

Milne et al., addressed how adverse childhood experiences impact the professional quality of life of residential care workers while considering resilience as a mediator for burnout, secondary traumatic stress and compassion satisfaction. This important study investigates how adverse childhood experiences (ACEs) impact the professional quality of life of care providers. The findings underscore the cumulative effects of personal trauma, examining how burnout, secondary traumatic stress, and compassion fatigue are mitigated by resilience. This research provides valuable insights into sustaining mental health professionals in demanding caregiving fields.

### Trauma, emotional dysregulation, and substance use in adolescents

The issue also addresses adolescents in outpatient clinic settings, with a study exploring PTSD, emotional dysregulation profiles, and substance use. By identifying key patterns and risk factors within this population, the research highlights the interconnectedness of trauma, behavioral regulation, and substance-related challenges, offering clinicians new pathways for intervention.

## Unifying themes: vulnerability, resilience, and innovation

Collectively, these contributions address some of the most pressing challenges in developmental psychopathology: The vulnerability of children and adolescents, especially those experiencing trauma, maltreatment, or systemic barriers to care. The mental health and well-being of caregivers, including the gendered nature of caregiving and its emotional toll. The need for resilience-based interventions, tailored support systems, and innovative strategies to address the ripple effects of trauma across individuals and systems. This issue is unique in its multi-faceted exploration of trauma—whether in child protection settings, outpatient clinics, or caregiving environments. It reflects not only the diversity of the challenges but also the resilience and innovative contributions of women researchers leading the field.

Through this Research Topic, we underscore the importance of amplifying women's voices in developmental psychopathology and mental health research. Their work shines a light on underserved populations, systemic barriers, and pathways for change—bringing us closer to equitable, trauma-informed care for all. We hope this collection inspires further research, policy change, and global efforts to promote gender equality in science, mental health, and developmental care.

## References

[1] Sá C Cowley S Martinez M Kachynska N Sabzalieva E. Gender gaps in research productivity and recognition among elite scientists in the US, Canada, and South Africa. PLoS One. (2020) 15(10):e0240903. 10.1371/journal.pone.0240903 33119671 PMC7595278

[2] Meho LI. The gender gap in highly prestigious international research awards, 2001–2020. Quant Sci Stud. (2021) 2(3):976–89. 10.1162/qss_a_00148

[3] Holman L Stuart-Fox D Hauser CE. The gender gap in science: how long until women are equally represented? PLoS Biol. (2018) 16(4):e2004956. 10.1371/journal.pbio.2004956 29672508 PMC5908072

[4] Sharma N Chakrabarti S Grover S. Gender differences in caregiving among family-caregivers of people with mental illnesses. World J Psychiatry. (2016) 6(1):7. 10.5498/wjp.v6.i1.7 27014594 PMC4804270

[5] Herman JL. Trauma and recovery: the aftermath of violence—from domestic abuse to political terror (Rev. ed., with a new epilogue). New York, NY: Basic Books (2015).

